# Developing the Canadian Indigenous Cognitive Assessment for Use With Indigenous Older Anishinaabe Adults in Ontario, Canada

**DOI:** 10.1093/geroni/igaa038

**Published:** 2020-08-31

**Authors:** Kristen Jacklin, Karen Pitawanakwat, Melissa Blind, Megan E O’Connell, Jennifer Walker, Andrine M Lemieux, Wayne Warry

**Affiliations:** 1 Department of Family Medicine and Biobehavioral Health, University of Minnesota Medical School Duluth; 2 Memory Keepers Medical Discovery Team - Health Equity, University of Minnesota Medical School Duluth; 3 Naandechige-Gamig Wikwemikong Health Centre, Ontario, Canada; 4 Department of Psychology, University of Saskatchewan, Saskatoon, Canada; 5 School of Rural and Northern Health, Laurentian University, Sudbury, Ontario, Canada

**Keywords:** Cognitive assessment, Cross-cultural care, Dementia, Indigenous

## Abstract

**Background and Objectives:**

Dementia is a growing public health issue for aging Indigenous populations. Current cognitive assessments present varying degrees of cultural, educational, and language bias, impairing their application in Indigenous communities. Our goal is to provide Anishinaabe communities in Canada with a brief cognitive test that can be administered within the community setting by community health workers or professionals. The purpose of this study was to adapt the Kimberly Indigenous Cognitive Assessment (KICA) for use as a brief cognitive test with Anishinaabe populations in Canada.

**Research Design and Methods:**

We used a community-based participatory research approach coupled with two-eyed seeing to provide an equitable space for Indigenous knowledge. Adaptation of the KICA was accomplished over 22 months using an iterative cycle of monthly consultations between an 11-member expert Anishinaabe language group (EALG) and the investigators, with ad hoc consultations with an Indigenous Elder, a community advisory council, and the KICA authors. Face validity was established with two pilot studies using cognitive interviewing with Indigenous older adults (*n* = 15) and a focus group consultation with local health professionals (*n* = 7).

**Results:**

Each question of the KICA was scrutinized by the EALG for cultural appropriateness, language and translation, and cultural safety. Every domain required adaptation to reflect cultural values, specificity of language, tone, and a culturally safe approach. Orientation, verbal comprehension and fluency, praxis, and naming domains required the most adaptations. The first pilot for face validity resulted in additional changes; the second confirmed satisfactory adaptation of all changes.

**Discussion and Implications:**

The research resulted in the new Canadian Indigenous Cognitive Assessment. The findings reveal important cultural and linguistic considerations for cross-cultural cognitive assessment in Indigenous contexts. This new culturally appropriate and safe brief cognitive test may improve case finding accuracy and lead to earlier diagnosis and improved dementia care for Indigenous peoples.

**Translational Significance:** This work resulted in a culturally safe brief cognitive assessment for Anishinaabe populations that, upon validation, can be used within multiple settings to help patients and caregivers document cognitive status and seek appropriate care.

The rates of Alzheimer’s disease and related dementias are not equal across ethnicities (Mayeda et al., 2016). Although reliable epidemiological data assessing dementia in Indigenous populations remain problematic (Warren et al., 2015), some Indigenous populations in North America have demonstrated higher prevalence and incidence of dementia than non-Indigenous populations (Jacklin et al., 2013; Mayeda et al., 2016). Understanding the source of this disparity requires careful and systematic evaluation of patients with memory complaints. While the need for culturally appropriate cognitive assessments for use with Indigenous populations in Western cultures such as Canada has been discussed for decades (Crossley, 2011; Hendrie et al., 1993; Jervis et al., 2007; Lanting et al., 2011), currently available cognitive assessments continue to present varying degrees of cultural, educational, and language bias. Potential bias for tests used with Indigenous peoples is a major threat to validity of test interpretation, limiting clinicians’ ability to accurately assess cognitive status with Indigenous peoples. Commonly used cognitive assessments such as the Mini-Mental State Examination (Folstein et al., 1975) or the Montreal Cognitive Assessment (Nasreddine et al., 2005) fail to account for culture, colonization, education, or health and social inequalities (O’Driscoll & Shaikh, 2017; Parker et al., 2007), which can produce false positives (Gasquoine, 2009). Cultural safety and appropriate cross-cultural care remain significant clinical barriers to equitable care for Indigenous populations and can impact dialogue between the provider and patient (Allen & Smylie, 2015; Greenwood et al., 2018). Clearly, there remains an urgent need to address cultural appropriateness and safety of cognitive testing tools.

To our knowledge, there is only one cognitive screening tool created using close collaboration with Indigenous peoples. To address their own practice gap, researchers in Australia used a community-based participatory research (CBPR) process to develop the Kimberly Indigenous Cognitive Assessment (KICA), which is grounded in the culture and language of Aboriginal communities in Central and Northern Australia. The KICA has revealed a high prevalence of dementia diagnoses in more than one Aboriginal community (Smith et al., 2008, 2009). Validation studies indicate that the KICA has good interrater reliability (α > 0.6), internal consistency (Cronbach’s α = 0.88), sensitivity (90.6%), and specificity (92.6%; LoGiudice et al., 2006). The KICA was reevaluated using the original 2006 sample and a second Indigenous Australian community, which again showed solid psychometric properties (Smith et al., 2009). The original Australian KICA tool has further developed into a suite of assessment tools comprising cognitive, informant, and functional assessments (Almeida et al., 2014; LoGiudice et al., 2004, 2011; Smith et al., 2016). The KICA has been extensively validated and modified for other regions in Australia and for Australian Indigenous peoples living in urban settings (Almeida et al., 2014; Radford et al., 2015; Smith et al., 2009). It was also adapted for Iranian adults (Ebrahimi et al., 2015), Brazilian Indigenous people (De Carvalho, 2016), and will be adapted for New Zealand Maori (Dudley, 2018a, 2018b). For these reasons, the KICA was a highly attractive option for adaptation to the North American context.

While attractive due to its cultural grounding and strong psychometric performance, there are significant differences between Australian and North American Indigenous cultures, for example, differences include geography, language, history, economy, policy, and culture. Research with Indigenous people in North America has shown that they perceive dementia symptoms differently (Jacklin, 2019), are diagnosed younger, and have shown dementia disparities compared with their non-Indigenous counterparts (Jacklin et al., 2013; Mayeda et al., 2016). Following consultation with Anishinaabe community advisors and the investigator team, the KICA was chosen for adaptation to a Canadian Indigenous (Anishinaabe) population due to its demonstrated validity in Indigenous populations and its rich CBPR history and respect for the sovereignty of Indigenous populations. The purpose of this study was to adapt the KICA for use as a brief cognitive test with Anishinaabe populations in Canada.

## Method

### Participants and Setting

**Figure 1.**
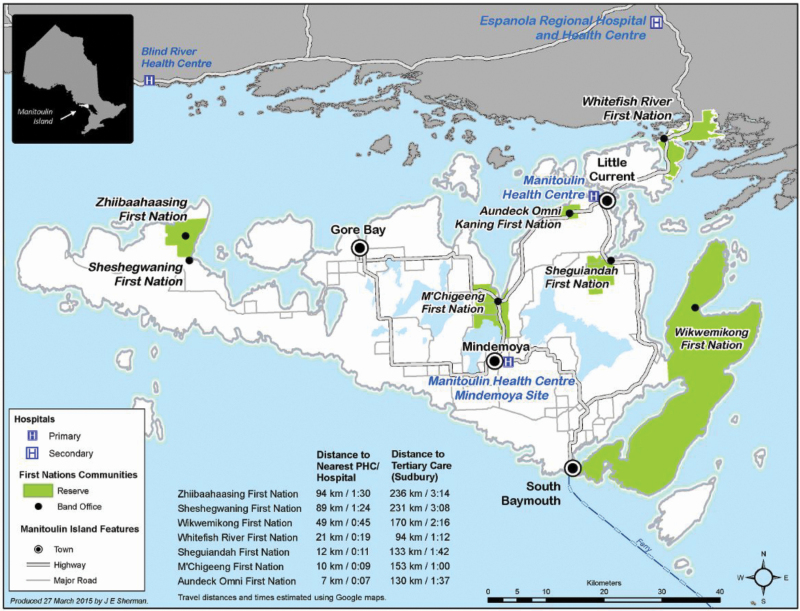
Manitoulin Island is located along the southern border of Ontario in Lake Huron. The location of the First Nations’ reserve is highlighted in green.

Mirroring the KICA development (LoGiudice et al., 2006; Smith et al., 2009), we implemented a CBPR study with the Anishinaabe First Nations communities in the Manitoulin Island region in northeastern Ontario. A critical component of this work was the incorporation of “two-eyed seeing,” an approach outlined by Mi’kmaw Elder Albert Marshall, where issues are considered from an Indigenous Knowledge and Western knowledge perspective (Bartlett et al., 2012). Seven First Nations communities, three First Nations Health Authorities, and an Aboriginal Health Access Centre participated. Manitoulin Island is situated in Lake Huron, Ontario, Canada and is approximately 3,107 km^2^. The seven First Nations (Figure 1) identify as Anishinaabe originally of the Three Fires Confederacy: Ojibwa, Odawa, and Potawatomi (Jacklin, 2009). There are approximately 4,500 First Nations inhabitants living on-reserve in the seven communities, and approximately 20% of those report an Indigenous language as their mother tongue with a range of 45.4% in Wikwemikong to 9.1% in Zhiibaahaasing (Government of Canada, Statistics Canada, 2017). Participant recruitment was for the following four primary functions: service on the Community Advisory Council (CAC; *n* = 8), service on the Expert Anishinaabe Language Group (EALG; *n* = 11), participation in face validity pilots (*n* = 15), and participation in health professional group interview (*n* = 7).

Recruitment to the CAC occurred first. Members for the CAC were recruited based on the recommendations from local Health Directors and leadership. The CAC comprises members from each of the seven First Nations who have either professional or lived experience with dementia (three men and five women). The role of the CAC is to advise the research team on all aspects of the research and ensure community voice and participation. The CAC recommended the methodology of establishing and working with the EALG.

Participants for the EALG included 11 older Anishinaabe adults (aged 55–86 years) from these seven First Nations (five men and six women). Members for the EALG were selected based on the recommendations from the CAC, Health Directors and leadership. Inclusion criteria included knowledge of the Anishinaabe language, understanding both traditional and contemporary changes to the language over time, ability to communicate in English and Anishinaabemwin, and respected community standing. The EALG’s role focused on adapting the KICA through discussion of the questions in the Anishinaabemwin language (Anishinaabemwin is the language of the Anishinaabe nation).

Participants for the two pilots to establish face validity were recruited by the community-based researcher (K. Pitawanakwat) and are best described as a purposeful sample. The first pilot included five women and five men; five of whom were “younger” older adults (aged 45–60 years) and five “older” older adults (aged 61–80 years). The lower age limit of 45 years reflects a lower age of onset and chronic illness related to aging in First Nations populations (Jacklin et al., 2013). Four of the participants completed the assessment in Anishinaabemwin and six in English. The second pilot involved five participants ranging in age from 45 to 70 (three men and two women), two completing the assessment in English, two in both English and Anishinaabemwin, and one completing the assessment in Anishinaabemwin.

Participants for the health professional group interview were drawn from health care providers servicing the seven First Nation communities and included nurses, an occupational therapist, a physician, and home care and personal support workers (*n* = 7). Participants were suggested by health directors who identified staff working with older adults or conducting cognitive assessments.

### Ethical Approval and Community Engagement

Research ethics approval was granted by the Laurentian University and the Manitoulin Anishinaabek Research Review Committee. Motions of approval were obtained from Chief and Council and Health Committees/Boards as deemed appropriate by the leadership. Ongoing community engagement included annual reports, community presentations, and biannual community newsletters.

### Data Collection and Analysis

This adaptation was an iterative process over a 22-month period from September 2015 to November 2017. The process involved the following two stages, each iterative: (a) the adaptation and (b) piloting and face validity.

### Adaptation

**Figure 2.**
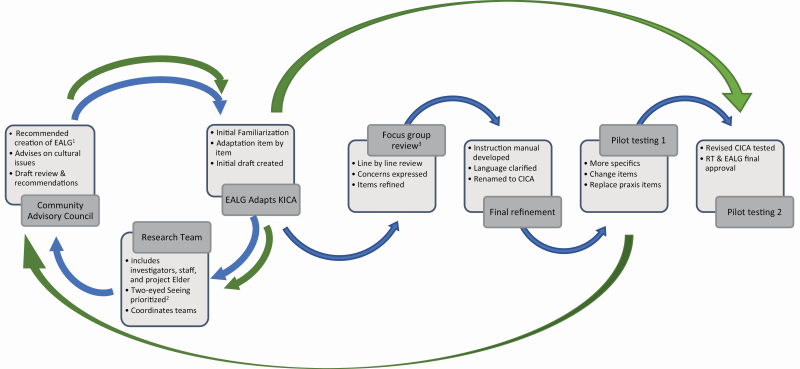
The iterative flow of communication during this project is depicted. ^1^EALG = Expert Anishinaabemwin Language Group consisting of older adults fluent in the Anishinaabe language and advanced in cultural and traditional knowledge. ^2^“Two-eyed Seeing” was the guiding principle of the research wherein the issue is examined from the strengths of Indigenous Knowledge as well as Western knowledge and ways of knowing. ^3^Focus group review completed via a panel of seven health professionals familiar with both dementia and the First Nations clients of Manitoulin region. The first path is represented by the blue arrows. The iterative feedback (second or more) path is represented by the green arrows.

Adaptation of the KICA involved each of the expert groups sharing information and requesting feedback or education from each other (Figure 2). Discussions were in English, with the exception of the EALG which was primarily in the Anishinaabe language and translated (K. Pitawanakwat). Each session was video recorded, edited, and organized by domain using the Camtasia Studio 8 software. In the case of the EALG, the audio recording and analytical notes of each meeting were translated into English and transcribed (K. Pitawanakwat). These transcriptions were shared with the research team, including a clinical neuropsychologist (M. E. O’Connell), to further assess the face validity of suggested revisions.

Ad hoc meetings with the authors of the KICA helped finalize the draft tool. Each adapted question was analyzed for clarity and face validity, sentence structure was simplified, and the rationale for each question was fully explained. This final draft version was then reviewed with the EALG to ensure the translation to Anishinaabemwin, and back translation to English was accurate. The CAC also reviewed the questions and approved it for piloting.

This stage also relied on ad hoc consultations with the project knowledge keeper and advisor Elder Jerry Otowadjiwan (see Author Note 1), who was part of the research team. Elder Otowadjiwan provided knowledge and insight into the cultural meaning of items under consideration for inclusion or exclusion as requested.

### Piloting—Face Validity

The draft assessment was first presented to a panel of local health care professionals. The 2-hr consultation was facilitated by K. Pitawanakwat, M. Blind, and K. Jacklin and was audio recorded and transcribed. Facilitator notes, postconsultation debriefing, and analytical notes from the audio recording were used to ascertain the appropriateness of the assessment questions from a provider perspective. Concerns raised by this group were shared with the EALG and investigators for consideration (Figure 2).

The first pilot involved 10 participants. The assessment was administered and then discussed in a debrief session with the participant. The assessment team for the pilot testing included a social worker, a personal support worker, and a registered nurse (K. Pitawanakwat). The assessment team recorded observations and comments, which were then brought to the research team, CAC, EALG, and Elder Jerry Otowadjiwan. Following the revisions, a second smaller pilot was conducted with five participants by the same assessment team using the same approach.

## Results

### Description of the Modifications and Participant Reactions

The adapted KICA was named the Canadian Indigenous Cognitive Assessment (CICA; Walker et al., 2020). The two pilot studies did not differ in the proportion of women (50% in Pilot 1; 40% Pilot 2) or inclusion of younger (vs. older) adults (50% in Pilot 1; 60% in Pilot 2; Fisher exact test, *p* > .10). The two pilot tests also had similar proportions of Anishinaabe speakers (40% Pilot 1; 60% Pilot 2; Fisher exact test, *p* > .10). Although the cognitive domains in the CICA remained the same as for those assessed in the KICA, adjustments were made to reflect the local context and culture, and nuances within the Anishinaabemwin language (Table 1).

**Table 1.** Original KICA and adapted CICA

Cognitive domain	KICA^a^	CICA^b^
Orientation	“Is this week pension/pay week?”	“What time of day is it?”
	“What time of the year is it now?”	“What time are we in right now; is it spring, summer, fall, or winter?”
	“What is the name of the community/place?”	“Do you know where you are right now? What is this place?”
Recognition/naming	“What do you call this?” followed by “What is this one for?” Comb	“What is the name of this?” “What is the name of these?” “What is the purpose of this?” “What is the purpose of these?” Spoon
	Cup (pannikin)	No change
	Matches	No change
	[Items hidden around room for recall by standing and placing items around the room]	[Items hidden around personal space, no standing or moving]
Memory Registration	“Tell me those things I showed you”	“Okay, now tell me what those things were.”
Verbal Comprehension	“Shut your eyes”	
	“First point to the sky and then point to the ground.”	“Pick up this piece of paper, fold it once, and give it back to me.”
Verbal Fluency	“Tell me the names of all the animals that people hunt.” *Acceptable prompts: “Anymore? What about in the air? In the water?”*	“Next we will ask you to name as many animals as you can in one minute, wild animals or domesticated animals.” [start written record] *Acceptable prompts after 15 seconds: “Are you able to think of any other animals? How about birds? How about fish?”*
Object Recall	“Where did I put the ≤*item from naming*≥?”	No change
Visual Naming	“I’ll show you some pictures. You tell me what they are. Remember these pictures for later on” [line drawing displayed] “What’s this?” Boy	“I will show you some drawings, like this leaf. (*Point to example drawing*). Tell me what is drawn. Your task is to remember these. I will ask you one other time. What is drawn here?” Tree
	Emu	Flower
	Billy/fire	Kettle/tea kettle
	Crocodile	Bird
	Bicycle	Horse “Remember, I will ask about these one other time.”
Frontal/Executive Function	“Look at this. Now you copy it.” (alternating crosses and circles)	“Copy these letters that you see here on this piece of paper.”
Free and Cued Recall	Free recall: “You remember those pictures I showed you? What were those pictures? Tell me.” Cued recall: “Which one did I show you before? One of three pictures.”	Free recall: “Do you remember those drawings I showed you? In any order, tell me what was drawn.” Cued recall: “Choose the one I showed you first, like the leaf [point to example drawing].”
Praxis	“Open this bottle and pour water into this cup”	“I have already loosened this small bottle. Pour however much you want into the small cup.”
	“Show me how to use a comb.”	“Show me how to use this spoon.”

*Note*: CICA = Canadian Indigenous Cognitive Assessment; KICA = Kimberly Indigenous Cognitive Assessment.

^a^ LoGiudice et al. (2004), see https://www.perkins.org.au/wacha/our-research/indigenous/kica/ for a copy of KICA-Cog. ^b^Items represent the final version. For the interim items in pilot Test 1, see text description.

### Orientation

The KICA begins with three questions related to time and place. The questions asked were the following: “Is this week pension/pay week?” “What time of the year is it now?” “What is the name of the community/place?” The EALG explained that the concept of time for Anishinaabe people is related to the different moon cycles. Each moon identifies what is happening or what the people should be preparing for in their region. Knowing the exact date or time is not important, given that these can be easily looked up on a calendar or clock. EALG suggestions for alternate questions revolved around seasons and activities associated with the weather or a particular month. Asking specifically “What month is it?” was acceptable for younger people, as they are taught the names of the month in school, but considered problematic for older traditional Anishinaabemwin speakers, who may not deem this type of information important. When the research team suggested the question “What season is it now?” the EALG thought it would be difficult to translate the word “season” into Anishinaabemwin and that the question would be less specific than “how does it feel outside right now.” The health professional panel of experts was concerned over standardization of answers and administration in institutional settings.

In the first pilot, we asked “What time are we in right now?” (Acceptable prompt: “is it spring, summer, fall, winter”) and “What is happening to the living natural changes outside right now?” (Acceptable prompt: “what are the trees doing right now?”). During the pilot, all participants had concerns about these time-related questions. The first question was considered to be vague and unclear by many English/non-Anishinaabemwin speaking participants who thought we were asking about time zones and not seasons. For the second question, four participants discussed the impacts of pollution and climate change on the natural environment. This brought forward feelings of sadness for these participants and disrupted the flow of the assessment. Both of these questions were further adapted and clarified and repiloted with five participants in Pilot 2 (Table 1). The final questions related to time are “What time of day is it right now?” and “What time are we in right now; is it spring, summer, fall or winter?”

For the third question in this domain (place identification), the EALG stressed the need to be specific in what was being asked. They advised that older traditional Anishinaabe people think about place in relation to kinship, ceremonies, and activities associated with the land, as well as language used to describe the historical changes to landscape. They also speculated that “place” may have a different meaning for younger people (an age category not included in this study). In order to capture the possible generational differences and specificity needed in the language, we revised the item and used the following questions: “Do you know where you are right now? What is this place?” There were no concerns recorded by the pilot study participants with this specific question.

### Recognition and Naming

This second domain was modified slightly from the original set of questions. In the KICA, a comb, pannikin (cup), and matches are used for naming. Participants name the object and describe the item’s use. The assessor then hides each item and reminds the participant to remember the location.

The EALG agreed that a comb, cup, and matches were common, easily recognizable objects but expressed concern with the assessor standing up and hiding the three objects around the room. They felt that these actions were inappropriate and could cause the person being assessed to feel like they were being made fun of or considered a “fool,” especially if the assessor placed objects in places where they typically do not belong. Once the team explained the purpose of hiding the objects was to detect other types of dementia, the EALG approved and the instructions were modified to have the assessor place each object around their personal space, without standing up or moving around the room, but out of the direct eyesight of the person being assessed. After the first pilot, the assessors realized that one of the objects, a comb, made some participants uncomfortable, especially in relation to the praxis item assessed in subsequent sections where they are asked to use the comb (see Praxis section). In order to address any cultural inappropriateness, the comb was replaced with a spoon. This is explained further within the praxis item.

### Registration

The language used in registration domain in the KICA, “tell me those things I showed you,” was considered to be too direct. The EALG stressed the need to soften the tone of the questions and allow ample time for participants to answer. The question was modified slightly and piloted as “Okay, now tell me what those things were?”

### Verbal Comprehension

The KICA uses the following two short commands to measure verbal comprehension: “Shut your eyes; First point to the sky and then point to the ground.” The EALG had concerns that the instructions were culturally inappropriate and potentially culturally unsafe to use with Anishinaabe people. First, the command “shut your eyes” was too direct and would require “softer” language if it were to be included. The EALG also shared that the person being assessed would require specific instructions on how long to keep their eyes closed or when to open them. This brought forth additional concerns related to historical trauma and mistrust of authority, evoking memories of Indian Residential Schools, and cultural safety in differential power situations such as a clinic. Pointing, within the second and third part of the command, was also culturally inappropriate. The EALG explained that in Anishinaabe cultures, there is a strong connection between the physical and spiritual world. Spirit is considered to be all around us and exists within all living things, including plants, animals, water, land, and other animate and inanimate objects. It is inappropriate and disrespectful to point with your fingers as there are spirits everywhere. The EALG also noted the instruction would not make sense if the assessment was conducted indoors, as a person would need to be outside in order to motion toward the sky or the ground if they were being truthful. Based on these concerns, this was changed to “Pick up this piece of paper, fold it once, and pass it back to me.” The close similarity to the three-stage command used in the Modified Mini-Mental State Examination (3MS) was discussed by members of the research team. The community researcher (K. Pitawanakwat) explained that the exact phrase used in the 3MS, “Take this paper with your left/right hand, fold it in half and hand it back to me” (McDowell, 2006), would be difficult to translate into Anishinaabemwin. For this reason, we retained the simpler language.

In the first pilot, participants who completed the assessment in Anshinaabemwin had difficulty understanding the phrase “fold it once” in the language due to the subtle differences in the pronunciation of “*skiganen*” (fold once) and “*paskiganen*” (fold more than once). Specific instructions were given to translators to state the command slowly and clearly. After the second pilot, the command in the CICA was changed from “pass it back to me” to “give it back to me” to reflect value of reciprocity in the language and soften the tone, as recommended by a participant in the second pilot.

### Verbal Fluency

In the KICA, the person being assessed was told they would be timed for 1 min and was given the following command: “Tell me the names of all the animals that people hunt.” The assessor using the KICA can use the following prompts: “Anymore? What about in the air? In the water?” to elicit further responses.

The EALG suggested that the question needed to be simplified, as many of the younger older adults may not hunt. They also expressed concern over older or traditional Anishinaabe people being timed and potentially feeling rushed. They explained that it is more important for older and traditional Anishinaabe people to take the mental time needed to find the correct and most exact words before speaking. The EALG reminded the research team that being meticulous with one’s words and speaking only the truth, is one of the guiding principles of the Seven Grandfather Teachings. While “younger” older adults may also live by these teachings, they may be more accustomed to the fast pace Western testing. After discussion around the need for the question to be specific and simple enough to be understood in both English and Anishinaabemwin, the question was revised to “Next we will ask you to name as many animals as you can, wild animals or domesticated animals.” The EALG recommended including the terms wild and domesticated to indicate that the inclusion of all animals that are commonly seen, not those that stay in the bush, as acceptable. The following statements were used to prompt participants after 15 s of silence and were accepted by all pilot participants: “Are you able to think of any other animals? How about birds? How about fish?” Using the phrase “anything more” as a prompt was rejected as not acceptable in the Anishinaabe language, as all animals are animate and not “things.”

During the first pilot, participants were not told that they had to answer the question within a certain amount of time in accordance to cultural protocols of not rushing an older or traditional Anishinaabe people. Assessors felt that participants who completed the assessment in Anishinaabemwin generally took longer to complete this question as the words used are more descriptive and generated additional stories. For the second pilot, the instructions were more explicit and participants were asked the following: “Next I (we) will ask you to name as many animals as you can in one minute, wild animals or domesticated animals. Start/please start now” (Table 1). The inclusion of the phrases “in one minute” and “start/please start now,” helped participants focus on the task and eliminated the questions on when to start and decreased the sharing of additional stories as was the case in the first pilot.

### Memory Recall

In the KICA, participants are asked where each object used in the second domain (comb, matches, and pannikin [cup]) was hidden. Due to the simplicity of these items, there were no major changes to the wording of the questions suggested by any of the collaborators. After the first pilot, the comb was replaced with a spoon. The participants were asked to report the location each of the three objects hidden on or near the assessor; “Where did I put the cup? Where did I put the spoon? Where did I put the matches?” One participant commented that women may answer this question more quickly than men because women need to know where everything in the household is.

### Visual Naming

In the KICA, participants are shown six pictures and are asked to name what is in each of the pictures. Participants are then asked to remember the pictures and name them later on. The EALG suggested changing the KICA pictures (boomerang, boy, emu, billy/fire, crocodile, and bicycle) to pictures or drawings of animate or inanimate objects common in Northern Ontario. They also discussed the nuances within the Anishinaabe language and the need to choose animate or inanimate objects that can only be described in one particular way. For example, the word “bicycle” can be described three different ways in the Anishinaabe language. In the Anishinaabe language, items are named using the following categories and purpose: function (includes action or movement), appearance (shape and color), where it lives, and what it eats. This is further complicated by individual family preferences in naming.

In the first pilot, participants were asked the following question, “I will show you some drawings. Tell me what is drawn. Your task is to remember these, I will ask you one other time.” The assessor would point to each drawing and ask the participant “What is drawn here?” After the participant named all the drawings, the assessor would ask the participant, “Do you think I could ask if you remember what I showed you?” The drawings included a sweater, as an example image, a tree, bird, tea kettle, hammer, and horse. The drawings were problematic for Anishinaabe culture and language as there was a mixture of animate and inanimate objects, and some drawings had multiple ways to describe them, such as sweater and hammer. Participants wanted the drawings of the tree and the bird to be simplified and look more realistic. The final CICA images included black and white drawings of a leaf (used as an example), a tree, a flower, a kettle or tea kettle (see Author Note 2), a bird, and a horse; all being animate. The question was also revised for clarity. Participants were asked, “I will show you some drawings, like this leaf. (*Point to example drawing*). Tell me what is drawn. Your task is to remember these. I will ask one other time.” After the participants name what is drawn, the assessor states, “Remember, I will ask about these one other time.”

### Frontal/Executive Function

The alternating crosses and circles used in the frontal/executive function domain within the KICA were retained in the CICA. The EALG thought the KICA instruction: “Look at this. Now you copy it” was too direct and cautioned that Anishinaabe peoples might be uncomfortable picking up a pencil if they have never gone to school or if they have passed all their signing responsibilities to someone else. Further, when translating the word “copy,” it meant “mark it down” such as making a sticky note to remind you of something. During the first pilot, the alternating crosses and circles were printed on the upper portion of an 8.5 × 11 sheet of paper with a line across the middle of the paper for participants to copy the alternating crosses and circles underneath. The assessors used the command “Show me how to copy this.” Most participants had comments or concerns centered on the lack of specificity in the instruction, for example, where to copy it and size of copied image. In the second pilot, a separate piece of paper was given to the participants to copy the image. Two participants suggested rewording the question to further clarify what was being asked. The instructions were changed to: “Copy these letters that you see here on this piece of paper.” Age, sex, or language was not related to whether an assessor noted comments for the frontal/executive function task.

### Free Recall and Cued Recall

These two domains ask participants to recall the images shown from the visual naming domain. In the free recall domain of the KICA, participants are asked, “You remember those pictures I showed you? What were those pictures? Tell me.” Again, the KICA language was considered to be too direct. In the CICA, the command was softened, and the instructions made more specific. For free recall, participants in the first pilot were asked, “Do you remember those drawings I showed you? (*Show example drawing*). What was drawn?” In Pilot 1, one of the participants asked the assessors if they needed to recall the drawings in the order they were shown. In Pilot 2, however, four out of the five participants asked the assessors if the recall needed to be in order. This resulted in the question being revised after the second pilot to: “Do you remember those drawings I showed you? In any order, tell me what was drawn.” Assessor notes from the first pilot did not appear to be related to sex or age of the participant, nor were there language differences in items recalled.

Similarly, the language used in the CICA for the cued recall question was softened to: “Choose the one I showed you at first (*one of three drawings on a page – show example first*).” While none of the participants had concerns with the language used in the question, the assessors further simplified the question wording for the second pilot to follow the example in visual naming domain. The question was revised to: “Choose the one I showed you first, like the [point to example drawing].” There were no comments or concerns noted in the second pilot.

### Praxis

The objects used in the Praxis domain were also used in naming and recognition, registration, and recall. In the KICA, participants are asked to pour water and comb hair using a water bottle and comb for props. During the adaptation process, the EALG expressed concern about older people with arthritis having trouble opening a bottle of water. The research team addressed this concern including an instruction to the assessor to loosen the lid of the water bottle. During the first pilot, participants questioned how much water to pour into the cup and wanted very specific instructions in order to complete the task. There was also concern about wasting the water that was poured. In these particular cases, the assessors would pour the water back into the bottle and inform the participant that it would be reused in other assessments. For the second pilot, the question was revised to: “I have already loosened this small bottle. Pour however much you want into the small cup.” There were no comments or concerns recorded.

The second praxis stimulus (comb) was used in the first pilot. Two younger participants had concerns over bringing the comb to their hair and one participant demonstrated the task on the interpreter’s hair. Rather than being related to hygiene, the research team learned from the EALG that this was likely related to the sacredness of hair within the Anishinaabe culture.

The research team met with Elder Jerry Otowadjiwan, to discuss alternatives. During alternative praxis stimuli discussions with the research team, a “key” was suggested but quickly rejected by Elder Otowadjiwan. This concern related to the potential as a trigger for traumatic memories. The Elder described Indigenous children being locked in rooms and closets by the nuns and priests at Indian Residential Schools, which operated from the 1870s through 1996 (Commission, 2015). Using a key as a stimulus could have acted as a trigger for disruptive traumatic memories. On the advice of Elder Otowadjiwan and the EALG, the research team settled on using a metal spoon as the stimulus. The question for the second pilot was, “show me how to use this spoon.”

### Implementation

The focus group with health professionals exposed concern over integration of the CICA with their existing assessment tools—some of which are provincially mandated for use. In Canada, cognitive test requirements vary by province and are mandated for prescribing guidelines and are mandated for use in home or long-term care. The focus group participants revisited the original intent of the CICA, which is to provide a culturally safe and brief cognitive assessment tool for community use to assist formal and informal caregivers with accessing additional health services and discussed the need for uptake of the tool at the local, regional, and provincial levels. The health care professionals also wanted additional information around training and expressed concern over having to carry physical objects with them in order to complete an assessment. As a result, the team produced a detailed CICA Guidebook, CICA Instruction Booklet, and four training videos: English only, Anishinaabemwin only, English–Anishinaabemwin translated, and American Sign Language (see CICA materials).

## Discussion

This research sought to adapt the KICA to a brief cognitive assessment appropriate for use with Anishinaabe older adults in Canada. We implemented a two-eyed seeing approach which relied on negotiations between Indigenous knowledge and Western biomedical knowledge to produce an assessment that meets the requirements of cultural safety and reliable and valid cognitive assessment that can be used by community health workers and registered professionals. The research has resulted in a modified version of the KICA that is culturally safe and appropriate for use with Anishinaabe people in Canada and is named the Canadian Indigenous Cognitive Assessment. The name of the CICA was proposed during broader conversations among the authors, CAC, EALG, and additional colleagues regarding the possible adaptation and uptake of the tool across different Canadian sites (Walker et al., 2020). The results reveal several important findings relevant to the adaptation and administration of cognitive screening tests with Indigenous peoples; these findings are primarily related to culture, cultural safety, language, and age and gender.

Anishinaabe values were central to many of the modifications made to the questions and sometimes conflicts between those values and necessities of the tests were left unresolved. One of the Anishinaabe values behind this is truth: When one speaks, they need to be able to speak the truth. Participants noted that when questions were not specific enough, were timed, or involved deception (hiding things), it was difficult to honor this value. This was especially noted in the questions related to orientation, registration, visual naming, and free/cued recall. Notably, these are also the cognitive domains which are some of the more sensitive items in the long-established screening tools (Coleman et al., 2017; Ericsson et al., 2017; Horton et al., 2015). Beyond the conflicts between Indigenous and Western culture and values, the research findings revealed that issues of cultural safety and systemic racism persist and continue to impact the care of older Anishinaabe adults. For example, the fact that the choice in cognitive screening tests is mandated by health authorities and is linked to access to supportive services. This necessitates additional steps of advocacy for uptake of culturally appropriate tools. Further, health care providers who may not fully or even partially understand Anishinaabe culture and values are largely in control of who is tested and what qualifies one for assessment. Yet, the development of the CICA was a community driven process precisely for these reasons, to allow communities to provide access and a degree of control over defining cognitive capacity in their population.

The EALG was crucial in revealing several cultural factors that go beyond a simple language translation. For example, though nominally related to aspects of language, the cultural and historical roots driving differences in the paralinguistics of the language (nonverbal communication) repeatedly became evident during the process. Most questions needed to be less direct and allow enough time for the participant to thoughtfully answer. Cultural values were important to consider in achieving less direct language. For example, the change from “pass it back” to “give it back” in verbal comprehension reflected the value of reciprocity—to give back.

We also found a high degree of precision in use of language was necessary for the Anishinaabemwin CICA, which appears to be related to the highly descriptive nature of the language. This was most evident in the orientation domain but was also noted by our experts in the areas of verbal comprehension, verbal fluency, free/cued recall, visual naming, frontal/executive function, and praxis. Importantly, in visual naming, we learned that the KICA pictures could not simply be replaced by those relevant to the culture and geography of the Anishinaabe, but rather careful consideration had to be made to whether objects were considered animate or inanimate and how many words for an object existed in Anishinaabemwin.

Cultural safety is achieved when the person receiving care perceives that the care was appropriate to their culture, that the practitioner is respectful and understanding of them as an Indigenous person, and when systemic barriers to appropriate care are absent (Webkamigad et al., 2019). With the help of the CAC, EALG, and Elder Otowadjiwan, the research team came to understand that ethical practice in test development demands that we work to ensure assessment tools are culturally safe and trauma informed. Furthermore, respecting and honoring the impacts of colonialism means that it is important to adapt the screening tool items in a way that respects cultural spiritual beliefs. Cultural adaptations occurred to respect Indigenous views of time and space, the values of reciprocity, humility, and respect and careful attention to relation worldviews among individuals, between humans and animals and between humans and the spiritual world. Taking a trauma informed approach (e.g., rejecting the use of keys as stimulus or requesting that clients close their eyes, which may make many older Indigenous people feel uncomfortable or unsafe) required the research team to be aware of, and acknowledge, the impacts of colonial polices and the systemic and institutional racism experienced by Indigenous people (Allen & Smylie, 2015; Greenwood et al., 2018).

In addition to the language, culture, and cultural safety considerations, this qualitative approach to test adaptation highlights the importance of careful tracking of both pilot participants and their assessors as a third important outcome of the study. Discussions of assessor observations (field notes, comments, and questions) revealed early clues that age and sex of the participant may have had important impacts in some, but not all, cognitive domain items. Consistent with existing evidence that receptive language skills and ability to follow multistage commands, such as those in the verbal comprehension item, are important clinical features of age-related cognitive impairment (Bell et al., 2019), this analysis showed that more assessor comments or questions occurred for older (61–80 years old) than younger (45–60 years old) participants. The findings suggested a sex difference in the registration item, with fewer notes for women than men indirectly suggesting more difficulty for men. This is also in line with existing literature showing that men tend to have greater difficulty with immediate memory recall (Sundermann et al., 2017; Wang & Tian, 2018). During the pilot, the assessors noticed that women answered the recall questions more quickly than men. One woman (participant) suggested this a gendered question, in that women would be better at answering since women need to know where everything in the home is located. This might reflect the well-documented female advantage in memory skills (Gurvich et al., 2018). While admittedly the current pilot test samples were small and meant for cognitive interview only, these data are sufficient to warrant careful tracking and inclusion of similar data analysis of sex and age differences in subsequent larger validation samples.

The limitations of this study include the choice to limit the study to a Canadian geographic region and small sample size of the pilot projects. Likewise, the restriction of images to animate stimuli (culturally defined as animate) could have significantly impacted multiple cognitive domains (naming, recall, and recognition). Similarly, the change from two short commands in the KICA to a longer three-stage command in the CICA may result in different demands on verbal comprehension, working memory, or praxis. Further validation studies are required to determine the impact of these modifications; nevertheless, it is important to emphasize that validity will occur for the CICA. Clinical decision guides (i.e., cutoff scores) will be developed for the CICA and cannot be inferred from the KICA. Despite these limitations, the current adaptation has notable strengths including the iterative nature involving three separate expert groups with unique strengths, the consultation with a practicing Clinical Neuropsychologist, ad hoc consultations with the authors of the original KICA, and the careful attention to more than just literation language translation. We would argue that our methodology and reliance on cultural and language experts resulted in a more fully culturally embedded assessment tool than is typical for most assessment adaptations to another culture.

In conclusion, this qualitative study demonstrated that the KICA was found to be acceptable and readily adapted to North American First Nations peoples. Careful planning of CBPR, multiple expert groups including language and cultural experts, and qualitative analysis led to the development of the CICA—a brief cognitive test that was accepted by both participants and the experts involved in its development. Analysis of assessor observations during piloting led to refinements in the instrument and identified sex and age differences that are consistent with existing Western literature on memory complaints and cognitive test performance. Future work will require reliability and validity studies for the CICA with attention to sex and age differences in total score achievement, as well as assessor–participant interactions and observations. Future directions include careful validation with an expanded sample of young (45–60 years old) and older (61–80 years old) Indigenous adults in the region of the adaptation, validation in other North American Indigenous populations, and an implementation study. The adaptation described here was an early, but crucial, collaborative step between Indigenous communities and academics in the goal of adapting a cognitive screening tool that will eventually be culturally responsive to diverse Indigenous populations in both rural and urban communities. The process will undoubtedly require not just test development and validation, but, if successful, it will lead to an understanding of which cognitive domains are most sensitive to cultural bias and the ways in which systemic changes could eliminate systemic racism within the clinical context. If successful, this future work will require test validation as well as documentation of system changes in care guidelines, provincial mandates, and institutional standards of care, and provider practice. Early evidence for validity of the CICA suggests that it is accurate in the identification of cognitive impairment in Indigenous peoples with and without dementia and with mild cognitive impairment versus dementia (O’Connell et al., 2020; Walker et al., 2020).

## Author Note

1.In the Anishinaabe culture, “Elder” is a title of honor reserved for those with extensive cultural experience, knowledge, and wisdom regardless of their age. While an Elder may also be an older adult, that is not a strict requirement and varies from community to community.

2.Kettle or tea kettle, as pointed out by EALG member Evelyn Roy, is animate. The shape of the kettle is similar to a duck and an accepted answer in the Anishinaabe language included “zhii zhiip kik” translated to duck pot or pail. The tea kettle is also used to boil water or make tea using plant-based medicines from the land around us. Tea is the most common way of ingesting medicines we need to live a good life and for good health. In this sense, the kettle or tea kettle is life giving and therefore listed as animate. Print and online Ojibwe Dictionaries also list the kettle or tea kettle as animate.
